# A sub-cubic time algorithm for computing the quartet distance between two general trees

**DOI:** 10.1186/1748-7188-6-15

**Published:** 2011-06-03

**Authors:** Jesper Nielsen, Anders K Kristensen, Thomas Mailund, Christian NS Pedersen

**Affiliations:** 1 Bioinformatics Research Centre (BiRC), Aarhus University, C. F. Møllers Alle 8, DK-8000 Aarhus C, Denmark; 2 Department of Computer Science, Aarhus University, Åbogade 34, DK-8200 Aarhus N, Denmark

## Abstract

**Background:**

When inferring phylogenetic trees different algorithms may give different trees. To study such effects a measure for the distance between two trees is useful. Quartet distance is one such measure, and is the number of quartet topologies that differ between two trees.

**Results:**

We have derived a new algorithm for computing the quartet distance between a pair of general trees, i.e. trees where inner nodes can have any degree ≥ 3. The time and space complexity of our algorithm is sub-cubic in the number of leaves and does not depend on the degree of the inner nodes. This makes it the fastest algorithm so far for computing the quartet distance between general trees independent of the degree of the inner nodes.

**Conclusions:**

We have implemented our algorithm and two of the best competitors. Our new algorithm is significantly faster than the competition and seems to run in close to quadratic time in practice.

## Background

The evolutionary relationship between a set of species is conveniently described as a tree, where the leaves represent the species and the inner nodes speciation events. Using different inference methods to infer such trees from biological data, or using different biological data from the same set of species, often yield slightly different trees. To study such differences in a systematic manner, one must be able to quantify differences between evolutionary trees using well-defined and efficient methods. One approach for this is to define a distance measure between trees and compare two trees by computing this distance. Several distance measures have been proposed, e.g. the symmetric difference [1], the nearest-neighbour interchange [2], the subtree transfer distance [3], the Robinson and Foulds distance [4], and the quartet distance [5]. Each distance measure has different properties and reflects different properties of the tree relationship.

**Figure 1:**
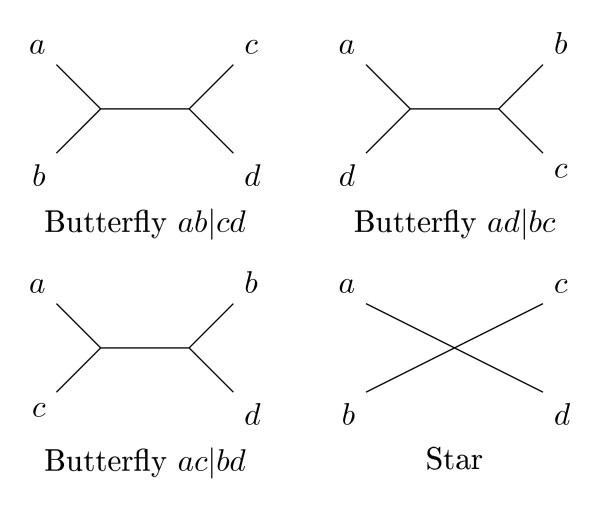
**Quartet topologies**. The four possible quartet topologies of species *a, b, c*, and *d*. For binary trees, only the butterfly quartets are possible.

For an evolutionary tree, the *quartet topology *of four species is determined by the minimal topological subtree containing the four species. The four possible quartet topologies of four species are shown in Figure 1. Given two evolutionary trees on the same set of *n *species, the *quartet distance *between them is the number of sets of four species for which the quartet topologies differ in the two trees.

Most previous work has focused on comparing *binary *trees and therefore avoided star quartets. Steel and Penny in [6] developed an algorithm for computing the quartet distance in time O(*n*^3^). Bryant *et al. *in [7] improved this result with an algorithm that computes the quartet distance in time O(*n*^2^). Brodal *et al.*, in [8], presented the currently best known algorithm that algorithm the computes the quartet distance in time O(*n *log *n*).

Recently, we have developed algorithms for computing the quartet distance between two trees of *arbitrary *degrees, i.e. trees that can contain star quartets. In [9] we developed two algorithms: the first algorithm runs in time O(*n*^3^) and space O(*n*^2^)--and is thus independent of the degree of the inner nodes--the second in time O(*n*^2^*d*^2^) and space O(*n*^2^), where *d *is the maximal degree of inner nodes in the trees--and thus depends on the degree of the nodes. The O(*n*^2^*d*^2^) was later improved to O(*n*^2^*d*) [10], and by taking an approach similar to the Brodal *et al. *[8] O(*n *log *n*) we developed a sub-quadratic algorithm in terms of *n *but at a significant cost in terms of *d*: O(*d*^9^*n *log *n*) [11].

In this paper we develop an O(*n*^2+*α*^) algorithm, where  and O(*n^ω^*) is the time it takes to multiply two *n *× *n *matrices. Using the Coppersmith-Winograd [12] algorithm, where *ω *= 2.376, this yields a running time of O(*n*^2.688^). The running time is thus independent of the degrees of the inner nodes of the input trees, and this is the first sub-cubic time algorithm with this property. Furthermore we have implemented the algorithm, along with two of the previous methods, and show experimentally that our new algorithm performs well in practice.

## Methods: A sub-cubic time and space algorithm

The quartet distance between two trees is the number of quartets where the quartet topology differs between the two trees, i.e. the number of quartets where one tree has the star topology and the other a butterfly topology, plus the number of quartets where the trees have a different butterfly topology. As observed in [9], the former--where one tree has the star topology and the other a butterfly topology--can be expressed in terms of the total number of butterflies in the two trees, the number of shared butterflies and the number of different butterflies: For trees *T *and *T'*, the number of different topologies due to one being a star and the other a quartet, diff*_S_*(*T*, *T'*), is given by(1)

where *B *is the number of butterflies in *T*, *B' *the number of butterflies in *T'*, shared*_B_*(*T*, *T'*) the number of quartets with the same butterfly topology in *T *and *T' *and diff*_B_*(*T'*) the number of quartets with different butterfly topologies in *T *and *T'*. Thus the quartet distance between *T *and *T' *is given by the expression(2)

Since, *B *= shared*_B_*(*T*, *T *) and *B' *= shared*_B_*(*T'*, *T'*), an algorithm for computing shared*_B_*(*T*, *T'*) and diff*_B_*(*T*, *T'*) gives an algorithm for computing the quartet distance between *T *and *T'*.

**Figure 2:**
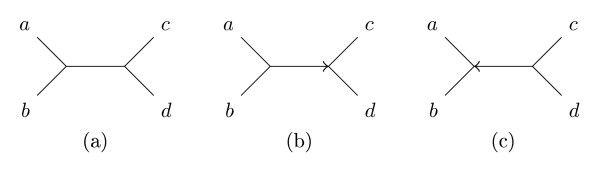
**Directed and undirected topologies**. An undirected quartet topology, (a), and the two directed quartet topologies, (b) and (c), it induces.

**Figure 3:**
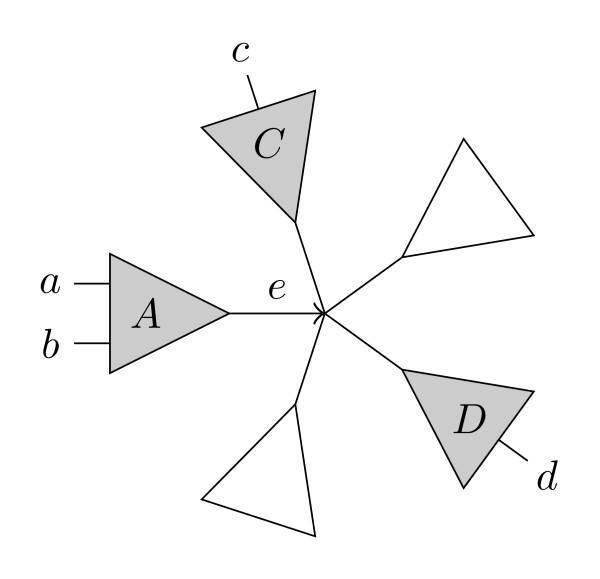
**A claim**. A *claim *. The claim  claims all ordered butterflies *ab *→ *cd *where *a, b *∈ *A *and *c *∈ *C,d *∈ *D *where *C *and *D *are two *different *subtrees in front of *e*.

Our approach to counting the shared and different quartets is based on *directed quartets *and *claims *[8,9]. An (undirected) butterfly quartet topology, *ab*|*cd *induces two directed quartet topologies *ab *→ *cd *and *ab *← *cd*, by the orientation of the middle edge of the topology, as shown in Figure 2. There are twice as many directed butterflies as undirected. If *e *= (*s_e_*, *t_e_*) is a directed edge from *s_e _*to *t_e _*we call *s_e _*the source of *e*, and *t_e _*the target. To each directed quartet, *ab *→ *cd*, we can uniquely associate the directed edge, *e *so that *a *and *b *are leaves in the subtree rooted at *s_e_*, and *c *and *d *are leaves in *different *subtrees rooted at *t_e_*, see Figure 3. We call such a tree substructure, consisting of a directed edge *e *with a subtree, A behind *e *and two distinct subtrees, *C *and *D*, in front of *e *a *claim*, written . We say that the edge *e claims *the directed quartet *ab *→ *cd*, and we also say that an edge *e *claims an undirected quartet *ab*|*cd *if it claims one of its directed quartets. Each (undirected) butterfly quartet defines exactly two directed butterfly quartets, and each directed quartet is claimed by exactly one directed edge; considering each claim and implicitly each directed butterfly claimed by the claim, we can examine each directed butterfly in a tree, or each undirected butterfly twice.

**Figure 4:**
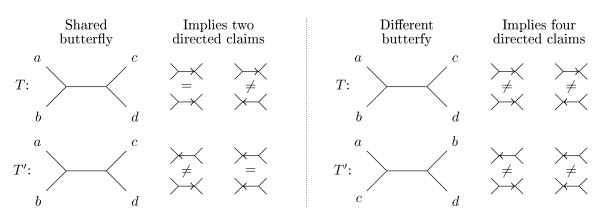
**Counting directed claims**. A shared butterfly induces two butterflies in each tree, which will give four pairs of claims, however the butterflies will only be identical in two of these pairs, thus a shared butterfly will be counted twice. A different butterfly also induces four pairs of claims, but since we are counting different butterflies all four will be counted. The way we count shared butterflies prevents the two different butterflies induced by the shared (undirected) butterfly from being counted.

The crux of the algorithm is to consider each pair of claims, one from each tree, and for each such pair count the number of shared and different directed butterflies claimed in the two trees. This way each shared butterfly is counted twice, and each different butterfly is counted four times, as shown in Figure 4. Dividing the counts by two and four, respectively, gives us shared*_B_*(*T*, *T'*) and diff*_B_*(*T*, *T'*).

### Preprocessing

Before counting shared and different butterflies, we calculate a number of values in two preprocessing steps. First, we calculate a matrix that for each pairs of subtrees *F *∈ *T *and *G *∈ *T' *stores the number of leaves in both trees, |*F *⋂ *G*|. This can be achieved in time and space O(*n*^2^) [7].

Next, for each pair of inner nodes, *v *∈ *T*, *v' *∈*T' *with sub-trees *F_i_*, *i *= 1,..., *d_v _*and *G_j_*, *j *= 1, ..., *d*_*v'*_, respectively, we calculate a matrix, *I*, such that *I*[*I*, *j*] = |*F_i _*⋂ *G_j_*|, and we calculate vectors of its row and column sums, and the total sum of its entries:(3)(4)(5)

Inspired by the sums (S.3) - (S.6) in Additional file 1 we calculate a matrix *I*', vectors of its row and column sums, the total sum of its entries, and some further values(6)(7)(8)(9)(10)(11)(12)(13)

Calculating the values in Eq. (3) - (13) can be done in O(*d_v_d_v'_*) for each pair of inner nodes (*v*, *v*') ∈ *T *× *T'*, giving a total time of . Finally, we need to calculate the following values:(14)

which takes time  for each pair of inner nodes, giving a total time of O(*n*^4^), if done naively. However, as we show in section 1 of Additional file 1 the values in Eq. (14) can be calculated faster if we precompute either and , or  and  depending on which pair of matrices is fastest to compute, where *I *is the *d_v _*× *d_v' _*matrix defined above. We thus calculate either Eq. (15) and (16), or Eq. (17) and (18), depending on which pair is fastest to calculate.(15)(16)(17)(18)

Calculating the values in Eq. (15) and (16) takes time  if padding the matrices to become square and with *ω *= 2.376 if using the Coppersmith-Winograd algorithm [12] for matrix multiplication, or time  if using naive matrix multiplication. Similarly, calculating the values in Eq. (17) and (18) takes time  or . Computing either  and , or  and , thus takes time .

### Counting shared butterfly topologies

**Figure 5:**
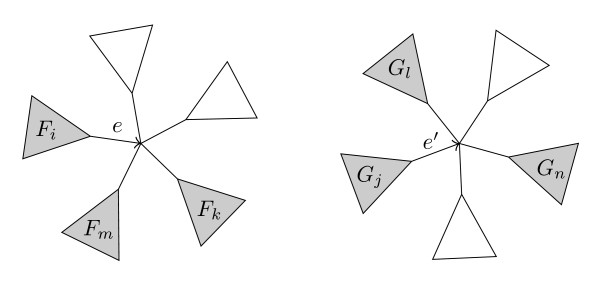
**Comparing two edges**. A pair of inner edges, *e *∈ *T*, *e' *∈ *T'*, where *F_i _*(*G_j_*) is the sub-tree behind *e *(*e'*) and *F_k_*, *k *≠ *i *(*G_l_*, *l *≠ *j*) the remaining subtrees of the node pointed to by *e *(*e'*). Highlighted are two claims, one from each tree.

**Figure 6:**
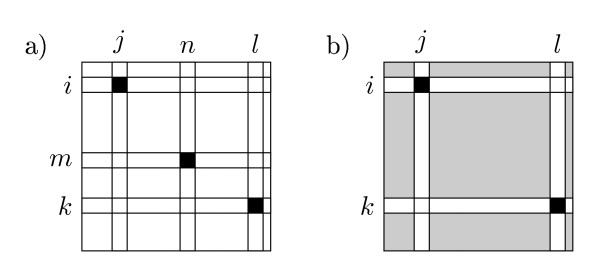
**Counting shared quartets**. Graphical illustration of the shared quartet expression, eq. (19). On the left, the matrix entries summed over are explicitly shown. On the right, the inner sum is implicitly shown. The sum of the greyed entries can be computed in constant time.

For each pair of inner edges, *e *∈ *T *and *e' *∈ *T'*, see Figure 5, we count the directed butterflies claimed by both *e *and *e'*. These are all on the form *ab *→ *cd*, where *a*, *b *∈ *F_i _*⋂ *G_j_*, *c *∈ *F_k _*⋂ *G_l _*and *d *∈ *F_m _*⋂ *G_n _*for some claims,  and , of *e *and *e'*. The total number of directed butterflies common for both *e *and *e' *is therefore given by the expression(19)

or the sum of  for all distinct entries in *I *but fixed (*i*, *j*), see Figure 6(a). We divide by two since we count each quartet twice, due to symmetry between the (*k*, *l*) and (*m*, *n*) pairs.

Notice, however, that the inner sum is simply the total sum of entries in *I*, *M*, except for the rows *i *and *k *and columns *j *and *l*, see Figure 6(b). Using(20)

and the precomputed values we can, as shown in section 2 of Additional file 1 rewrite the expression in Eq. (19) to(21)

which can be computed in time O(1), if the referenced matrices have been precomputed. Thus we can compute all shared directed butterflies in total time O(*n*^2^). Dividing by two, we get the number of shared undirected butterflies.

### Counting different butterfly topologies

**Figure 7:**
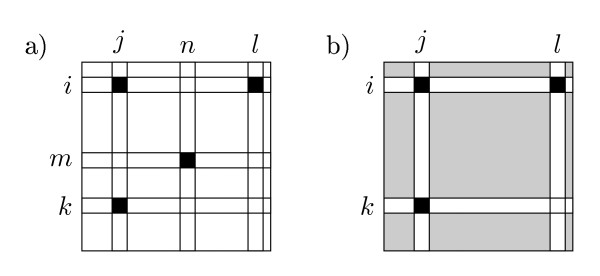
**Counting different quartets**. Graphical illustration of the different quartet expression, eq. (22). On the left, the matrix entries summed over are explicitly shown. On the right, the inner sum is implicitly shown. The sum of the greyed entries can be computed in constant time.

Counting the number of different butterflies in the two trees is done similar to counting the number of shared butterflies. As before, we consider a pair of inner edges, *e *∈ *T *and *e' *∈ *T'*. The quartets claimed by both *e *and *e'*, but with different butterfly topology, are on the form *a *∈ *F_i _*⋂ *G_j_*, *b *∈ *F_i _*⋂ *G_l_*, *c *∈ *F_k _*⋂ *G_j _*and *d *∈ *F_m _*⋂ *G_n _*for some claims  and . The number of butterflies claimed by both *e *and *e' *but with different topology is therefore given by(22)

or the sum of *I*[*I*, *j*] · *I*[*I*, *l*] · *I *· [*k*, *j*] *I*[*m*, *n*] for all distinct entries in *I *but fixed (*I*, *j*), see Figure 7. In this case there is no need to divide by any normalizing constant, since there are no symmetries between *k *and *m *or between *l *and *n*.

As before, the inner sum can be expressed as in Eq. (20), and using the precomputed values we can, as shown in section 3 of Additional file 1 rewrite the expression in Eq. (22) as(23)

or(24)

depending on whether we have precomputed  and , or  and . We can thus compute Eq. (22) in time O(1) for each pair of inner edges *e *∈ *T *and *e' *∈ *T' *giving a total time of O(*n*^2^) to compute different directed, and thus different undirected, butterfly topologies in the two trees.

To get the actual number of different butterflies we have to divide by four.

### Time analysis

The running time of the algorithm is dominated by the time  it takes to compute either  and , or  and , for each pair of nodes *v *∈ *T *and *v' *∈ *T'*. Let O(*n^ω^*) be the time it takes to multiply two *n *× *n *matrices. In section 4 of Additional file 1 we show that the running of our algorithm is O(*n*^2+*α*^), where . Using the Coppersmith-Winograd algorithm [12] for matrix multiplication, where *ω *= 2.376, this yields a running time of O(*n*^2.688^).

## Results

**Figure 8:**
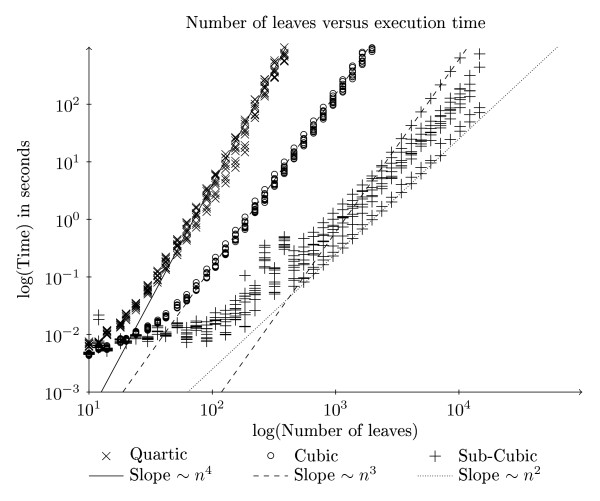
**Comparison of algorithms**. Plotted are our new algorithm compared to previously known O(*n*^3^) and O(*n*^4^) algorithms. In a log-log plot *x^b ^*becomes a straight line with the slope determined by *b*. The lines in the plot are not regression lines, but are inserted to help the reader judge the time complexity of our implementations.

**Figure 9:**
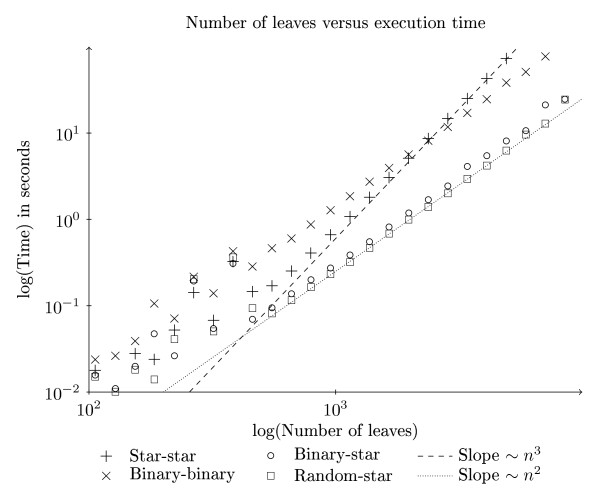
**Comparison of tree topologies**. This plot shows the two best and the two worst pairs of tree topologies, for our new algorithm only. In a log-log plot *x^b ^*becomes a straight line with the slope determined by *b*. The lines in the plot are not regression lines, but are inserted to help the reader judge the time complexity of our implementation.

We have implemented our new algorithm and, for comparison, the O(*n*^3^) and O(*n*^4^) algorithms [9] for general trees. We chose those algorithm instead of those from [10,11], because the running time of those algorithms are dependent on the degree of the nodes, while a major feature of our new algorithm is that it has a good asymptotical running time independent of the degree of the nodes. For matrix multiplication we link to a BLAS library, and expect that to choose the most efficient algorithm for matrix multiplication. In our experiments the vecLib library from Mac OS X is used. We have run benchmarks with trees with ten leaves up to trees with almost 15, 000 leaves. For each size, trees were generated in four different ways: general trees, binary trees, star trees and trees with one node of degree  surrounded by degree 3 nodes. The code that generated the trees is available in Additional file 2.  For each of the ten possible combinations of topologies, one pair of trees were randomly generated, and the time used for the computation of the quartet distance was measured and plotted. Our experiments were run on a Mac-Pro with two Intel quad-core Xeon processors running at 2.26 GHz and with 8 GB RAM. As seen in Figure 8 the implementation of our new algorithm is significantly faster than the implementations of the competing algorithms, on trees with many leaves. In the worst cases our algorithm approaches O(*n*^3^) which is expected if the BLAS implementation uses the O(*n*^3^) matrix multiplication algorithm. Indeed Figure 9 shows that the slowest of our runs are on two star-shaped trees, where we need to multiply two *n *× *n *matrices and where the time-complexity of the matrix multiplication algorithm is most important. However, in most cases our algorithm seems to be close to quadratic execution time, even though it apparently uses an asymptotically slow matrix multiplication algorithm.

## Conclusion

We have derived, implemented and tested a new algorithm for computing the quartet distance. In theory our algorithm has execution time O(*n*^*α*+2^), where . With current knowledge of matrix multiplication this is O(*n*^2.688^). If an algorithm for matrix multiplication in time O(*n*^2^) is found this would make our algorithm run in time O(*n*^2.5^). Experiments on our implementation shows it to be fast in practice, and that it can have a running time significantly better than the theoretical upper bound, depending on the topology of the trees being compared.

## Availability

The software is available from http://www.birc.au.dk/software/qdist. It has been tested on Ubuntu Linux and Mac OS X.

## Competing interests

The authors declare that they have no competing interests.

## Authors' contributions

JN, TM and CP developed the algorithm. AK implemented the algorithm. AK and JN benchmarked and evaluated the algorithm. JN, TM and CP wrote the paper. All authors read and approved the paper.

## Supplementary Material

Additional file 1 **Supplementary material containing mathematical derivations that are too tedious for the main text**. Click here for file

Additional file 2 The python script used to generate the random trees for the experiments. Click here for file
