# Supplementary material for: A sub-cubic time algorithm for computing the quartet distance between two general trees
Source: Algorithms Mol Biol. 2011 Jun 3;6:15. doi: 10.1186/1748-7188-6-15 (PMC3141660; doi:10.1186/1748-7188-6-15)
Supplement: Additional file 1 — Supplementary material containing mathematical derivations that are too tedious for the main text. [file 1748-7188-6-15-S1.PDF]

Supplementary material for: A sub-cubic time algorithm for computing the quartet distance between two general trees

Jesper Nielsen*[1,2], Anders Kabell Kristensen[2], Thomas Mailund[1] and Christian N.S. Pedersen[1,2]

[1]Bioinformatics Research Centre (BiRC), Aarhus University, C. F. Møllers Allé 8, DK-8000 Aarhus C, Denmark
[2]Department of Computer Science, Aarhus University, Åbogade 34, DK-8200 Aarhus N, Denmark

Email: Jesper Nielsen*- jn@birc.au.dk; Anders Kabell Kristensen - anders.kabell.kristensen@gmail.com; Thomas Mailund - mailund@birc.au.dk; Christian N.S. Pedersen - cstorm@birc.au.dk;

*Corresponding author

1 Computing $I'''[i,j]$

If I''_1 and I'''_1 are available, we can compute $I'''[i,j]$ as:

$$\begin{aligned}
\sum_{k\neq i}\sum_{l\neq j} I[i,l]I[k,j]I[k,l] &= \sum_{k\neq i} I[k,j]\sum_{l\neq j} I[i,l]I[k,l]\\
&= \sum_{k\neq i} I[k,j]\left(I''_1[i,k] - I[i,j]I[k,j]\right)\\
&= \left(\sum_{k\neq i} I[k,j]I''_1[i,k]\right) - \left(\sum_{k\neq i} I[k,j]^2 I[i,j]\right)\\
&= I'''_1[i,j] - I[i,j]I''_1[i,i] - \left(I[i,j]\sum_{k\neq i} I[k,j]^2\right)\\
&= I'''_1[i,j] - I[i,j]I''_1[i,i] - I[i,j]\left(C'''[j] - I[i,j]^2\right) \qquad \text{(S.1)}
\end{aligned}$$

If instead I''_2 and I'''_2 are available, we can compute $I'''[i,j]$ symmetrically:

$$\begin{aligned}
\sum_{k\neq i}\sum_{l\neq j} I[i,l]I[k,j]I[k,l] &= \sum_{l\neq j} I[i,l]\sum_{k\neq i} I[k,j]I[k,l]\\
&= \sum_{l\neq j} I[i,l]\left(I''_2[j,l] - I[i,j]I[i,l]\right)\\
&= \left(\sum_{l\neq j} I[i,l]I''_2[j,j]\right) - \left(\sum_{l\neq j} I[i,l]^2 I[i,j]\right)\\
&= I'''_2[i,j] - I[i,j]I''_2[j,j] - \left(I[i,j]\sum_{l\neq j} I[i,l]^2\right)\\
&= I'''_2[i,j] - I[i,j]I''_2[j,j] - I[i,j]\left(R'''[i] - I[i,j]^2\right) \qquad \text{(S.2)}
\end{aligned}$$

2 Rewriting Eq. (19) to Eq. (21)

Expanding Eq. (20) into Eq. (19) in the main paper yields:

$$\frac{1}{4}\binom{|F_i \cap G_j|}{2} \sum_{k\neq i}\sum_{l\neq j} |F_k \cap G_l| \sum_{m\neq i,k}\sum_{n\neq j,l} |F_m \cap G_n|$$

$$= \frac{1}{4}\binom{I[i,j]}{2} \Bigg($$

$$\left(\sum_{k\neq i}\sum_{l\neq j} I[k,l](M - R[k] - C[l] + I[k,l])\right) + \tag{S.3}$$

$$\left(\sum_{k\neq i}\sum_{l\neq j} I[k,l](I[i,j] - R[i] - C[j])\right) + \tag{S.4}$$

$$\left(\sum_{k\neq i}\sum_{l\neq j} I[k,l]I[i,l]\right) + \tag{S.5}$$

$$\left(\sum_{k\neq i}\sum_{l\neq j} I[k,l]I[k,j]\right)\Bigg) \tag{S.6}$$

Using the values introduced in the section on preprocessing, we can rewrite the sums (S.3) – (S.6) as:

$$\begin{aligned}\sum_{k\neq i}\sum_{l\neq j} I[k,l](M - R[k] - C[l] + I[k,l]) &= \sum_{k\neq i}\sum_{l\neq j} I'[k,l] \\ &= M' - R'[i] - C'[j] + I'[i,j]\end{aligned} \tag{S.7}$$

$$\begin{aligned}\sum_{k\neq i}\sum_{l\neq j} I[k,l](I[i,j] - R[i] - C[j]) &= (I[i,j] - R[i] - C[j])\sum_{k\neq i}\sum_{l\neq j} I[k,l] \\ &= (I[i,j] - R[i] - C[j])(M - R[i] - C[j] + I[i,j])\end{aligned} \tag{S.8}$$

$$\begin{aligned}\sum_{k\neq i}\sum_{l\neq j} I[k,l]I[i,l] &= \sum_{l\neq j} I[i,l] \sum_{k\neq i} I[k,l] \\ &= \sum_{l\neq j} I[i,l](C[l] - I[i,l]) \\ &= R''[i] - I[i,j](C[j] - I[i,j])\end{aligned} \tag{S.9}$$

$$\begin{aligned}\sum_{k\neq i}\sum_{l\neq j} I[k,l]I[k,j] &= \sum_{k\neq i} I[k,j] \sum_{l\neq j} I[k,l] \\ &= \sum_{k\neq i} I[k,j](R[k] - I[k,j]) \\ &= C''[j] - I[i,j](R[j] - I[i,j])\end{aligned} \tag{S.10}$$

which gives that Eq. (19) can be rewritten as Eq. (21).

3 Rewriting Eq. (22) to Eq. (23) or (24)

Expanding Eq. (20) into Eq. (22) in the main paper yields:

$$\begin{aligned}
&|F_i \cap G_j| \sum_{k\neq i}\sum_{l\neq j} |F_i \cap G_l||F_k \cap G_j| \sum_{m\neq i,k}\sum_{n\neq j,l} |F_m \cap G_n| \\
&= I[i,j] \sum_{k\neq i}\sum_{l\neq j} I[i,l]I[k,j] \sum_{m\neq i,k}\sum_{n\neq j,l} I[m,n] \\
&= I[i,j]\Bigg(\\
&\quad \left(\sum_{k\neq i}\sum_{l\neq j} I[i,l]I[k,j](M - R[i] - C[j] + I[i,j])\right) + && \text{(S.11)} \\
&\quad \left(\sum_{k\neq i}\sum_{l\neq j} I[i,l]I[k,j](I[k,j] - R[k])\right) + && \text{(S.12)} \\
&\quad \left(\sum_{k\neq i}\sum_{l\neq j} I[i,l]I[k,j](I[i,l] - C[l])\right) + && \text{(S.13)} \\
&\quad \left(\sum_{k\neq i}\sum_{l\neq j} I[i,l]I[k,j]I[k,l]\right)\Bigg) && \text{(S.14)}
\end{aligned}$$

Again we use the values introduced in the section on preprocessing to rewrite the sums (S.11) – (S.13) as:

$$\begin{aligned}
&\sum_{k\neq i}\sum_{l\neq j} I[i,l]I[k,j](M - R[i] - C[j] + I[i,j]) \\
&\qquad = (M - R[i] - C[j] + I[i,j])(R[i] - I[i,j])(C[j] - I[i,j]) \qquad \text{(S.15)}
\end{aligned}$$

$$\begin{aligned}
\sum_{k\neq i}\sum_{l\neq j} I[i,l]I[k,j](I[k,j] - R[k]) &= \sum_{k\neq i} I[k,j](I[k,j] - R[k]) \sum_{l\neq j} I[i,l] \\
&= \sum_{k\neq i} I[k,j](I[k,j] - R[k])(R[i] - I[i,j]) \\
&= (R[i] - I[i,j]) \sum_{k\neq i} I[k,j](I[k,j] - R[k]) \\
&= (R[i] - I[i,j])(I[i,j](R[i] - I[i,j]) - C''[j]) \qquad \text{(S.16)}
\end{aligned}$$

$$\begin{aligned}
\sum_{k\neq i}\sum_{l\neq j} I[i,l]I[k,j](I[i,l] - C[l]) &= \sum_{l\neq j} I[i,l](I[i,l] - C[l]) \sum_{k\neq i} I[k,j] \\
&= \sum_{l\neq j} I[i,l](I[i,l] - C[l])(C[j] - I[i,j]) \\
&= (C[j] - I[i,j]) \sum_{l\neq j} I[i,l](I[i,l] - C[l]) \\
&= (C[j] - I[i,j])(I[i,j](C[j] - I[i,j]) - R''[i]) \qquad \text{(S.17)}
\end{aligned}$$

The sum (S.14) is $I'''[i,j]$. Replacing the sums (S.11) – (S.13) with Eq. (S.15) – (S.17) and (S.14) with either (S.1) or (S.2) shows that Eq. (22) can be rewritten as Eq. (23) or Eq. (24), respectively.

4 Time analysis

In the main paper we claim that the running time of the algorithm is $\mathrm{O}\big(n^{2+\alpha}\big)$, where $\alpha = \frac{\omega-1}{2}$, if it takes time $\mathrm{O}(n^{\omega})$ to multiply two $n \times n$ matrices. For each pair of nodes $(v, v') \in T \times T'$ we need to compute either I_1'' and I_1''', or I_2'' and I_2''', which takes time $\mathrm{O}\big(\min(\max(d_v, d_{v'})^{\omega}, d_v^2 d_{v'}, d_v d_{v'}^2)\big)$. It is this computation that dominates the running time. To show the claimed running time, we split the pairs of nodes $(v, v') \in T \times T'$ into four disjoint sets

$$\begin{aligned}
P_1 =& \{(v, v') \in T \times T' \mid d_v \le d_{v'} \wedge d_v \le d_{v'}^{\alpha}\} \\
P_2 =& \{(v, v') \in T \times T' \mid d_v \le d_{v'} \wedge d_v > d_{v'}^{\alpha}\} \\
P_3 =& \{(v, v') \in T \times T' \mid d_v > d_{v'} \wedge d_v^{\alpha} \ge d_{v'}\} \\
P_4 =& \{(v, v') \in T \times T' \mid d_v > d_{v'} \wedge d_v^{\alpha} < d_{v'}\}
\end{aligned}$$

which can be analysed independently. Preprocessing for all pairs of nodes in P_1, where $d_v \le d_{v'}^{\alpha} \le n^{\alpha}$, takes time:

$$\begin{aligned}
\mathrm{O}\left(\sum_{(v,v') \in P_1} \min(\max(d_v, d_{v'})^{\omega}, d_v^2 d_{v'}, d_v d_{v'}^2)\right) &= \mathrm{O}\left(\sum_{(v,v') \in P_1} d_v^2 d_{v'}\right) \\
&\le \mathrm{O}\left(\sum_{v \in T \mid d_v \le n^{\alpha}} \sum_{v' \in T'} d_v^2 d_{v'}\right) \\
&= \mathrm{O}\left(\sum_{v \in T \mid d_v \le n^{\alpha}} d_v^2 \sum_{v' \in T'} d_{v'}\right) \\
&= \mathrm{O}\left(\sum_{v \in T \mid d_v \le n^{\alpha}} d_v^2 n\right) \\
&= \mathrm{O}\left(\sum_{v \in T \mid d_v \le n^{\alpha}} d_v d_v n\right) \\
&\le \mathrm{O}\left(\sum_{v \in T \mid d_v \le n^{\alpha}} d_v n^{\alpha} n\right) \\
&= \mathrm{O}(n n^{\alpha} n) \\
&= \mathrm{O}\big(n^{2+\alpha}\big)
\end{aligned}$$

Preprocessing for all pairs of nodes in P_2, where $d_{v'}^{\alpha} < d_v \le n$, takes time:

$$\begin{aligned}
\mathrm{O}\left(\sum_{(v,v')\in P_2} \min(\max(d_v, d_{v'})^{\omega}, d_v^2 d_{v'}, d_v d_{v'}^2)\right) &= \mathrm{O}\left(\sum_{(v,v')\in P_2} d_{v'}^{\omega}\right) \\
&= \mathrm{O}\left(\sum_{v'\in T'} \sum_{v\in T \mid d_{v'}^{\alpha} < d_v \le d_v'} d_{v'}^{\omega}\right) \\
&= \mathrm{O}\left(\sum_{v'\in T'} d_{v'}^{\omega} \sum_{v\in T \mid d_{v'}^{\alpha} < d_v \le d_{v'}} 1\right) \\
&= \mathrm{O}\left(\sum_{v'\in T'} d_{v'}^{\omega} \sum_{v\in T \mid d_{v'}^{\alpha} < d_v \le d_{v'}} \frac{d_v}{d_v}\right) \\
&\le \mathrm{O}\left(\sum_{v'\in T'} d_{v'}^{\omega} \sum_{v\in T \mid d_{v'}^{\alpha} < d_v \le d_{v'}} \frac{d_v}{d_{v'}^{\alpha}}\right) \\
&= \mathrm{O}\left(\sum_{v'\in T'} d_{v'}^{\omega-\alpha} \sum_{v\in T \mid d_{v'}^{\alpha} < d_v \le d_{v'}} d_v\right) \\
&= \mathrm{O}\left(\sum_{v'\in T'} d_{v'}^{\omega-\alpha} n\right) \\
&= \mathrm{O}\left(\sum_{v'\in T'} d_{v'} d_{v'}^{\omega-\alpha-1} n\right) \\
&\le \mathrm{O}\left(\sum_{v'\in T'} d_{v'} n^{\omega-\alpha-1} n\right) \\
&= \mathrm{O}\left(\sum_{v'\in T'} d_{v'} n^{\omega-\alpha}\right) \\
&= \mathrm{O}\left(n n^{\omega-\alpha}\right) \\
&= \mathrm{O}\left(n^{1+\omega-\frac{\omega-1}{2}}\right) \\
&= \mathrm{O}\left(n^{2+\frac{\omega-1}{2}}\right) \\
&= \mathrm{O}\left(n^{2+\alpha}\right)
\end{aligned}$$

Since the cases P_3 and P_4 are symmetric to P_1 and P_2 respectively, the total running time is $\mathrm{O}(n^{2+\alpha})$.
